# Gut Microbiota Dysbiosis Aggravates *Mycoplasma gallisepticum* Colonization in the Chicken Lung

**DOI:** 10.3389/fvets.2021.788811

**Published:** 2021-11-30

**Authors:** Jian Wang, Xueping Chen, Jichang Li, Muhammad Ishfaq

**Affiliations:** ^1^College of Veterinary Medicine, Shanxi Agricultural University, Jinzhong, China; ^2^College of Veterinary Medicine, Northeast Agricultural University, Harbin, China; ^3^College of Computer Science, Huanggang Normal University, Huanggang, China

**Keywords:** gut microbiota, immune response, *Mycoplasma gallisepticum*, colonization, chickens

## Abstract

*Mycoplasma gallisepticum* (MG) is the pathogen that causes chronic respiratory diseases in chickens. Gut microbiota plays an important role in maintaining body health and resisting respiratory infection, but the correlation between gut microbiota and MG infection is poorly defined. Therefore, in this study, the correlation between gut microbiota and MG infection was explored by disturbing gut microbiota in chickens with antibiotic cocktail. The results showed that the gut microbiota dysbiosis impairs pulmonary immune response against MG infection. It has been noted that MG colonization in the lung was significantly increased following gut microbiota dysbiosis, and this could be reversed by intranasally administrated toll-like receptor 2 (TLR2) ligand, recombinant chicken IL-17 protein or recombinant chicken granulocyte-macrophage colony-stimulating factor (GM-CSF) protein. In addition, the levels of short-chain fatty acids (SCFAs) and vitamin A were significantly reduced in gut microbiota dysbiosis group, however, butyric acid or vitamin A as feed additives promoted MG clearance in the lung of gut microbiota dysbiosis group via increasing TLR2/IL17/GM-CSF and host defense peptides genes expression. The present study revealed an important role of gut microbiota in the defense against MG colonization in the lung of chicken.

## Introduction

*Mycoplasma gallisepticum* (MG) is one of the most common causes of chronic respiratory disease in chicken that remains a major issue to the poultry farmers and caused huge economic losses to the poultry production such as excessive feed consumption, reduced weight gain, low egg production, and hatchability (1, 2). Macrolides and tetracyclines are commonly used to control MG infection (3). However, the long-term use of antibiotics can increase *Mycoplasma* and bacterial resistance, which complicates the therapy of MG infection and other bacterial infections in chickens (3). Therefore, clarifying the key factors that regulate susceptibility or colonization resistance to MG could help in developing novel intervention strategies.

Large microbial communities (the gut microbiota) colonizing in the gastrointestinal tract of human and animal, have an extensive influence on host physiology during homeostasis and disease (4). Gut microbiota not only plays a role in the resistance to intestinal infection, but also have a systemic effect on antibacterial defenses at sites outside the intestine, such as lung (4). It has been determined that gut microbiota can play a crucial role in the defense against influenza virus, *Klebsiella pneumonia*, and *Streptococcus pneumoniae* through the “gut-lung axis” (4, 5). Although the understanding of the “gut-lung axis” is only just beginning, but new evidences showed that strategies to target gut microbiota have the potential to control lung diseases. For example, oral administration of *Lactobacillus* or *Bifidobacterium* can reduce the incidence of respiratory infections and reduce the critical degree of the disease (6). A gut commensal bacterium known as segmented filamentous bacterium, was also reported to promote *Staphylococcus aureus* clearance in lung (7). In addition, gut microbiota dysbiosis chickens showed significant defects in immune response to avian pathogenic *Escherichia coli* and gut microbiota metabolite acetate that significantly inhibited the lung inflammatory injury and reduced bacterial load in lung tissues induced by APEC infection (8). Therefore, we hypothesized that gut microbiota is a vital factor that modulate susceptibility or colonization resistance to MG. This study confirmed that the gut microbiota plays a positive role to protect against MG colonization in lung. Our research provides the theoretical basis for the new potential control strategy of MG infection.

## Materials and Methods

### Chickens

Specific pathogen-free (SPF) white Leghorn chickens were purchased from the Laboratory Animal Center of Harbin Veterinary Research Institute of Chinese Academy of Agricultural Sciences (Harbin, China). All chickens were housed in a sterile isolation chamber (IPQ-Type 3 negative pressure isolator; Shanxi Agricultural University, Taigu, China), and food and water were provided *ad libitum*. All animal experiments were performed according to the guidelines of Laboratory Animal Ethics Committee of Shanxi Agricultural University (Shanxi, China) in compliance with Laboratory animal-Guideline for ethical review of animal welfare (GB/T35892-2018, National Standards of the People's Republic of China).

### Study Design

**Figure 1:**
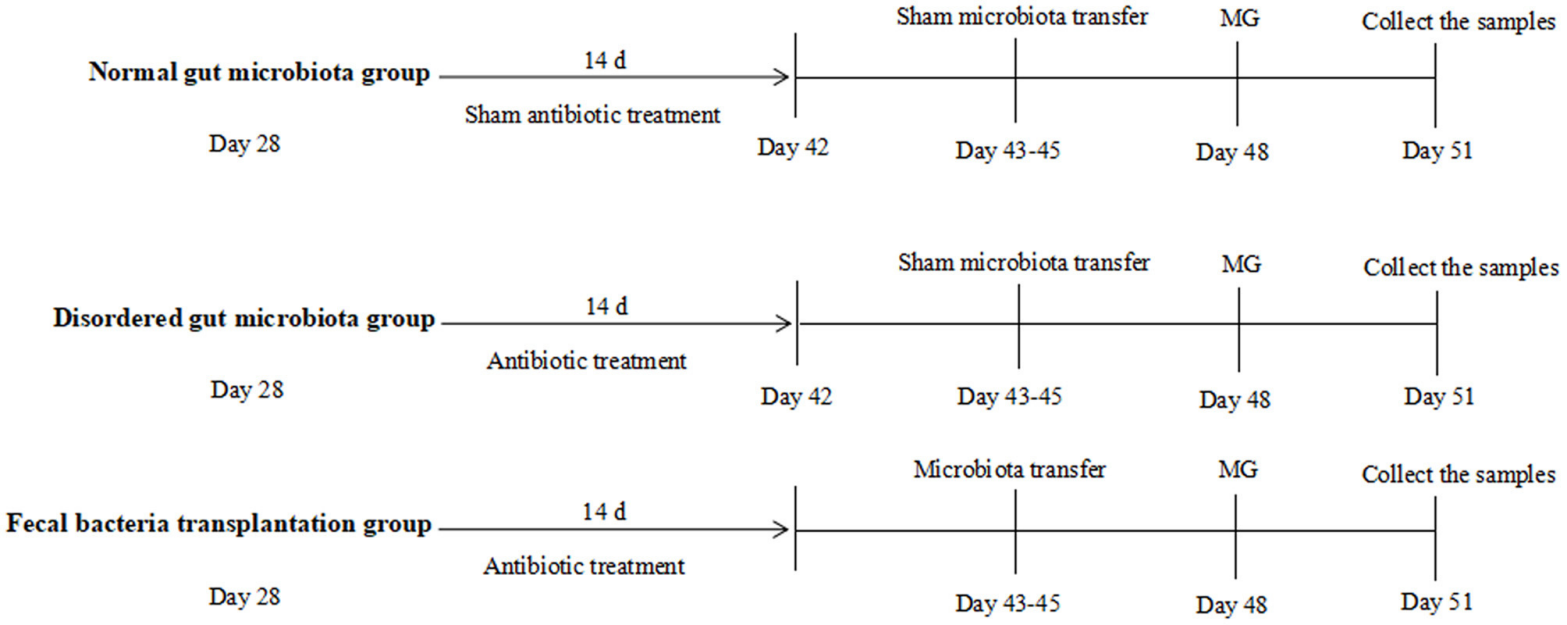
Schematic outline of the experimental protocol used in the present study. Details showed in the Materials and method section.

Chickens were randomly assigned to experimental groups at day 28. As showed in Figure 1, normal gut microbiota group (N group) chickens were given normal drinking water, chickens were challenged by MG at day 48, samples were collected at day 51; disordered gut microbiota group (D group) chickens received a cocktail of antibiotics (ampicillin 1 g/L, neomycin sulfate 1 g/L, metronidazole 1 g/L, and vancomycin 0.5 g/L) in drinking water for 2 weeks to disturb gut microbiota as descried previously (4), chickens were challenged by MG at day 48, samples were collected at day 51; fecal bacteria transplantation group (F group) received a cocktail of antibiotic as described above for 2 weeks, then received gut microbiota transplantation from N group chickens at day 45 and challenged by MG at day 48, samples were collected at day 51. Details of TLR2 ligand, recombinant proteins, vitamin A and butyric acid were applied as showed in Figure legends.

### Reagents

Ampicillin, neomycin sulfate, metronidazole, vancomycin, vitamin A, and sodium butyrate were purchased from Solarbio life science (Beijing, China). TLR2 ligands Pam3CSK4 (P3C) was purchased from InvivoGen (Hongkong, China). Recombinant chicken GM-CSF protein (rGM-CSF) and recombinant chicken IL-17 protein (rIL-17) were purchased from Abcam (Cambridge, MA, UK).

### MG Infection

MG strain (R_low_) was kindly donated by Professor Xin Jiuqing of Harbin Veterinary Research Institute, Chinese Academy of Agricultural Sciences. MG were grown in a modifed Hayfick medium, consisting of 0.1% nicotinamide adenine dinucleotide (NAD), 0.05% penicillin, 20% FBS, 0.05% thallium acetate and 10% freshly prepared yeast extract, as described in a previous study (9). Chickens were infected as described previously with some modification (2), a 0.3 mL aliquot solution containing 3 × 10^9^ CCU MG R_low_ were injected in the air sacs at indicated time points showed in Figure 1.

### Fecal Bacteria Transplantation

Fecal bacteria transplantation was performed as described previously (10). Fecal samples were weighed and homogenized (1:2 wt/vol) in sterile PBS, the fecal suspension vigorously mixed and the supernatant was collected. Chickens from F group were orally inoculated with 1 mL of fecal suspension at indicated time as showed in Figure 1.

### Quantification of MG Colonization in the Lung

To detect the extent of MG infection, absolute abundance of MG was measured by quantitative PCR (qPCR) using a recombinant plasmid consist of a cloned *mgc2* gene of MG to establish a standard curve as previously described (11). The recombinant plasmid was diluted by a gradient of 10 times to establish a standard curve. The genomic DNA were extracted by using Bacterial Genomic DNA Extraction Kit (Solarbio life science, Beijing, China) according to manufacturer's recommended procedures, and *mgc2* gene was amplified from genomic DNA by PCR and confirmed by Sanger sequence (Genewiz, Suzhou, China). The primers are: *mgc2*-F: 5′-TTGGGTTTAGGGATTGGGATT; *mgc2*-R: 5′ -CCAAGGGATTCAAC CATCTT. The reaction mixture was 1 × SYBR Green PCR Master Mix (Takara, Dalian, China), 0.2 mM of each forward and reverse primer, and 1 μL template DNA in a total reaction volume of 25 μL. The PCR reaction conditions are as follows: 1 cycle of 5 min at 95°C followed by 95°C for 10 s, 55°C for 30 s, 72°C for 60 s for 38 cycles and a final extension at 72°C for 5 min.

### Quantitative Real-Time PCR (qRT-PCR) Analysis

The procedure was carried out as described previously (2). Briefly, total RNA was extracted using Trizol reagent (Life Technologies, Grand Island, NY, USA) following the manufacturer's instructions. One microgram of total RNA were reverse transcribed using the Transcriptor First Strand cDNA Synthesis Kit (Transgen, Beijing, China). The gene expression levels were detected using real-time PCR with a SYBR premix Ex Taq kit (Transgen, Beijing, China) on a Roche 480 real-time PCR system thermocycler. Each sample was analyzed in triplicates. Target gene expression was normalized to β-actin. The primers are shown in Table 1.

**Table 1:** Primers used for qRT-PCR.

**Genes**	**Primer sequences**	**References**
GM-CSF	F:CCGTTTCAGGAACCAGAGAG R: GTCTGGCTGCTGGACATTTT	(12)
IL-17	F: CCATTCCAGGTGCGTGAACT R: TTTCTTCTCCAGGCGGTACG	(13)
IL-6	F:CAAGGTGACGGAGGAGGAC R: TGGCGAGGAGGGATTTCT	(14)
TLR2	F:TCTGCAAAAGGCTGTGAACCT R: CCAAACGAGTCCTCATCTATGGA	(15)
IL-2	F:TCTGGGACCACTGTATGCTCT R: ACCGACAAAGTGAGAATCAATCA	(14)
IL-18	F:GGAATGCGATGCCTTTTG R: ATTTTCCCATGCTCTTTCTCA	(14)
IL-13	F:CCAGGGCATCCAGAAGC R: CAGTGCCGGCAAGAAGTT	(14)
IL-1β	F:TGGGCATCAAGGGCTACA R: TCGGGTTGGTTGGTGATG	(14)
TGF-β	F:CGGGACGGATGAGAAGAAC R: CGGCCCACGTAGTAAATGAT	(14)
AvBD3	F:ATGCGGATCGTGTACCTGCTC R: CAGAATTCAGGGCATCAACCTC	(16)
AvBD9	F:GCAAAGGCTATTCCACAGCAG R:AGCATTTCAGCTTCCCACCAC	(16)
AvBD10	F:TGGGGCACGCAGTCCACAAC R:ATCAGCTCCTCAAGGCAGTG	(16)
IL-4	F:TCTTCCTCAACATGCGTCAG R:TGGTGGAAGAAGGTACGTAGG	(13)
IL-10	F:CATGCTGCTGGGCCTGAA R: CGTCTCCTTGATCTGCTTGATG	(17)
TLR4	F:ACCTCAATGCGATGCACTCT R: AGTCCGTTCTGAAATGCCGT	(13)
TNF-α	F:AGTGCTGTTCTATGACCGCC R:CGCTCCTGACTCATAGCAGA	(13)
IL-8	F:CTGCGGTGCCAGTGCATTAG R: GCACACCTCTCTTCCATCC	(17)
IL-12	F:TGGTCCACGCTTTGCAGA T R: AAG GTTAAGGCGTGGCTTCTT A	(18)
IL-15	F:TCTGTTCTTCTGTTCTGAGTGAT R:AGTGATTTG CTTCTGTCTTTGGT	(18)
β-actin	F:CATCTATGAAGG CTACGCCCT R:GCTTCTCCTTGATGTCACGCACAA	(12)

### 16S Sequencing and Data Analysis

DNA was extracted from the feces using Tiangen Stool DNA extraction kit (Tiangen, Beijing, China) according to the manufacturer's instructions. 16S rRNA gene V3 and V4 regions were amplified, and the primers are as follows: 341F (5′-ACTCCTACGGGAGGCAGCAG-3′) and 806R (5′ -GGACTACHVGGGTWTC TAAT-3′). 16S sequencing and data analysis were performed as previously described (19). Data has been uploaded to SRA under the accession number PRJNA601545.

### Short-chain Fatty Acids Quantification

SCFAs quantification was performed using a GC-MS method as previously described (19). The acetic acid, propionic acid, butyric acid standards (Solarbio, Beijing, China) was diluted by a gradient of 10 times of the working solution using ultrapure water. Fifty microliter working solution or 50 μL serum incubated with 400 μL NaCl and 50 μL of 3 mM hydrochloric acid sodium chloride solution, then the samples were exposed to ultrasonic treatment for 1 h at 4°C. The samples were added 1 mL of ice ether by vortex mixing for 15 min and centrifuged at 12,000 g for 15 min at 4°C. The supernatant was precipitated with an anhydrous sodium sulfate by vortex mixing for 5 min and was centrifuged at 5,000 g for 10 min at 4°C. The supernatant was used for SCFAs quantification using an Agilent 7890B/5977A GC-MS equipped with an Agilent HP-FFAP (25 m × 0.50 mm × 0.32 μm) column in a SIM mode.

### Vitamin Quantification

Vitamin quantification was performed using a LC-MS method as stated previously (20). Hundred microliter serum of each group was transferred to a tube, added 1 mL of extract solvent (acetonitrile-methanol-water, 2:2:1), the mixture were vortexed for 45 s, homogenized at 45 Hz for 5 min, and sonicated for 5 min at 4°C. The procedure of homogenation and sonication were repeated twice, and incubation at −20°C for 1 h and centrifuged at 12,000 g at 4°C for 10 min. The supernatant was transferred to a new tube, then dried under nitrogen, added 100 μl of 50% acetonitrile/water. The mixture was centrifuged at 12,000 g for 15 min at 4°C. The supernatant was transferred to an autosampler bottle for UHPLC-MS/MS (ultrahigh-performance liquid chromatography coupled to tandem mass spectrometry) analysis.

### Statistical Analysis

The data are expressed as mean ± SD and analyzed using GraphPad Prism 8.0 (GraphPad Software). Significant differences between the two groups were evaluated by two-tailed unpaired Student's *t* test or Mann-Whitney *U* test for samples that were not normally distributed. Statistical significance was determined by two-way ANOVA with Tukey tests for multiple-group comparisons. The level of significance was set at *P* < 0.05.

## Results

### Gut Microbiota Dysbiosis Aggravates MG Colonization

**Figure 2:**
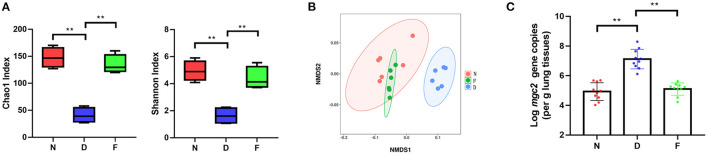
The effects of gut microbiota dysbiosis on MG colonization in lung. **(A)** Alpha diversity of gut microbiota in each group (*n* = 6), fecal samples were collected in the 48 day of age without MG challenge. **(B)** Non-metric multidimensional scaling (NMDS) of the gut microbiota composition on the operational taxonomic unit (OTU) level based on the Bray-Curtis distance (*n* = 6), fecal samples were collected in the 48 day of age without MG challenge. **(C)** *Mgc2* gene copies in chicken lung after MG infection (*n* = 6). ^**^Indicates *P* < 0.01.

In order to explore the role of gut microbiota in MG infection, we first dysbiosed gut microbiota by feeding antibiotics cocktail for 2 weeks. The results showed that antibiotic treatment significantly changed the diversity and composition of gut microbiota (Figures 2A,B, *P* < 0.01). In addition, Fecal bacteria transplantation could restore gut microbiota diversity and composition in F group (Figures 2A,B, *P* < 0.01). Subsequent experiments were performed as follows.

MG isolation culture is time-consuming; therefore, we used a quantitative PCR detection method of MG (11). In D group, there was a significantly increased MG colonization in the lung at 3 days post MG inoculation compared to N group (Figure 2C, *P* < 0.01); there was a significantly decreased MG colonization in the lung at 3 days post MG inoculation in F group compared to D group (Figure 2C, *P* < 0.01). These results determined that gut microbiota dysbiosis aggravated MG colonization in lung.

### Gut Microbiota Dysbiosis Impairs Pulmonary Immune Response

**Figure 3:**
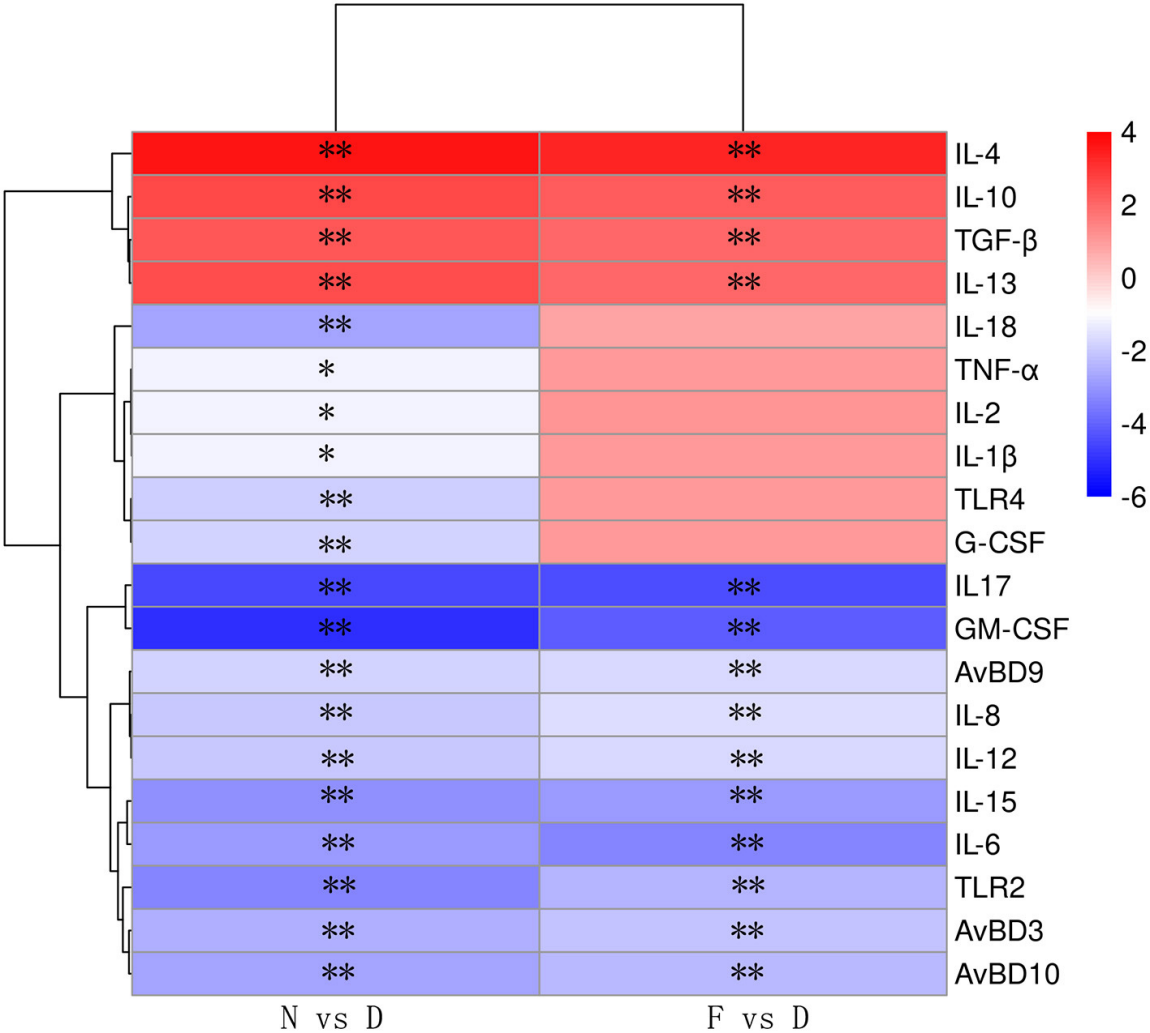
The relative mRNA expression levels of immune-related genes are shown using the indicated pseudo color scale (*n* = 6). Lung samples were collected in the 48 day of age without MG challenge. ^**^Indicates *P* < 0.01.

**Figure 4:**
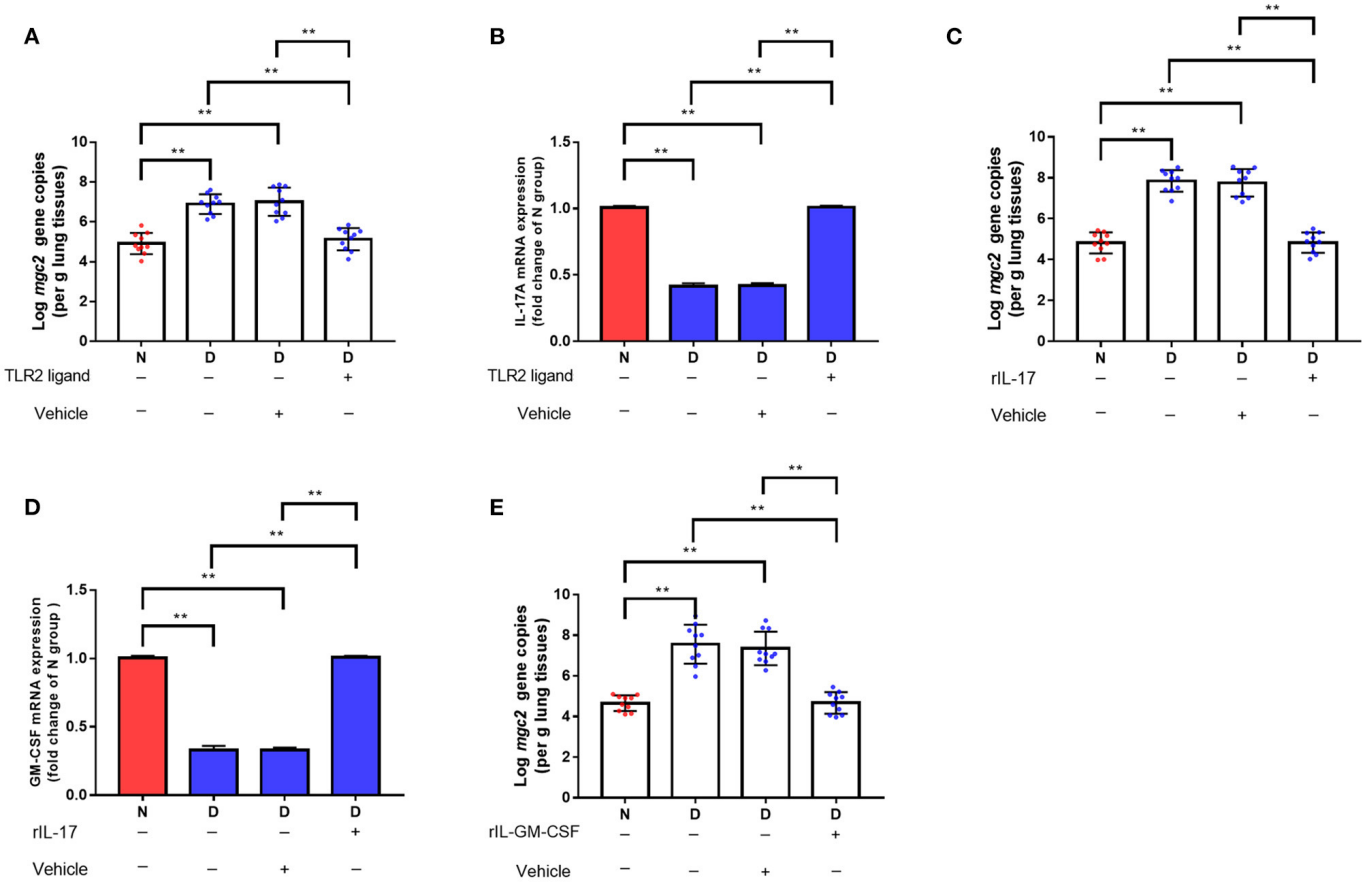
The effects of TLR2, TL-17, and GM-CSF on MG colonization in lung of gut microbiota dysbiosis group. **(A)** *Mgc2* gene copies in chicken lung after TLR2 ligand administration and MG infection (*n* = 10). **(B)** IL-17 mRNA expression levels in chicken lung after TLR2 ligand administration and MG infection (*n* = 6). **(C)** *Mgc2* gene copies in the chicken lung after rIL17 administration and MG infection (*n* = 10). **(D)** GM-CSF mRNA expression levels in chicken lung after rIL17 administration and MG infection (*n* = 6). **(E)** *Mgc2* gene copies in chicken lung after rGM-CSF administration and MG infection (*n* = 10). Indicated groups were intranasally administered TLR2 ligand (P3C, 50 μg) or 0.05 μg rIL17 or 0.05 μg rGM-CSF 1 h prior to MG infection at indicated dates showed in Figure 1. Values were expressed as means ± SD. ^**^Indicates *P* < 0.01.

We hypothesized that the decreased clearance of MG in D group might be related to the weakened immune response in lung. Therefore, a series of immune related genes were detected by qRT-PCR. Compared to N group and F group, D group exhibited significantly reduced pro-inflammatory genes and host defense peptide genes expression (Figure 3, *P* < 0.01), and significantly increased anti-inflammatory genes expression (Figure 3, *P* < 0.01). Among these different expression genes, TLR2, IL17 and GM-CSF have been confirmed to be associated with MG infection (9, 21), thus, the role of TLR2, IL17 and GM-CSF in gut microbiota dysbiosis aggravates MG colonization were explored. The results showed that intranasal administration of TLR2 ligand in D group promoted MG clearance (Figure 4A, *P* < 0.01). Consistent with the results of MG clearance, intranasal administration of TLR2 ligand increased IL-17 mRNA expression levels in D group (Figure 4B, *P* < 0.01). IL-17 plays a significant role in defense infection (4). To examine the role of IL-17 in regulating lung antibacterial immunity, chickens were administered rIL17 concomitant with MG inoculation. The rIL17 could promote MG clearance in D group (Figure 4C, *P* < 0.01). The rIL17A also could promote GM-CSF mRNA expression in D group (Figure 4D, *P* < 0.01). GM-CSF is critical signaling molecule in the innate response to respiratory infection as well as a member of the IL-17 signaling pathway. To examine the role of GM-CSF in regulating lung antibacterial immunity, chickens were administered rGM-CSF concomitant with MG inoculation and confirmed that the rGM-CSF could promote MG clearance in D group (Figure 4E, *P* < 0.01). Taken together, the above results showed that gut microbiota dysbiosis impairs pulmonary immune response against MG infection.

### Gut Microbiota Dysbiosis Alters SCFAs and Vitamin A Metabolism

**Figure 5:**
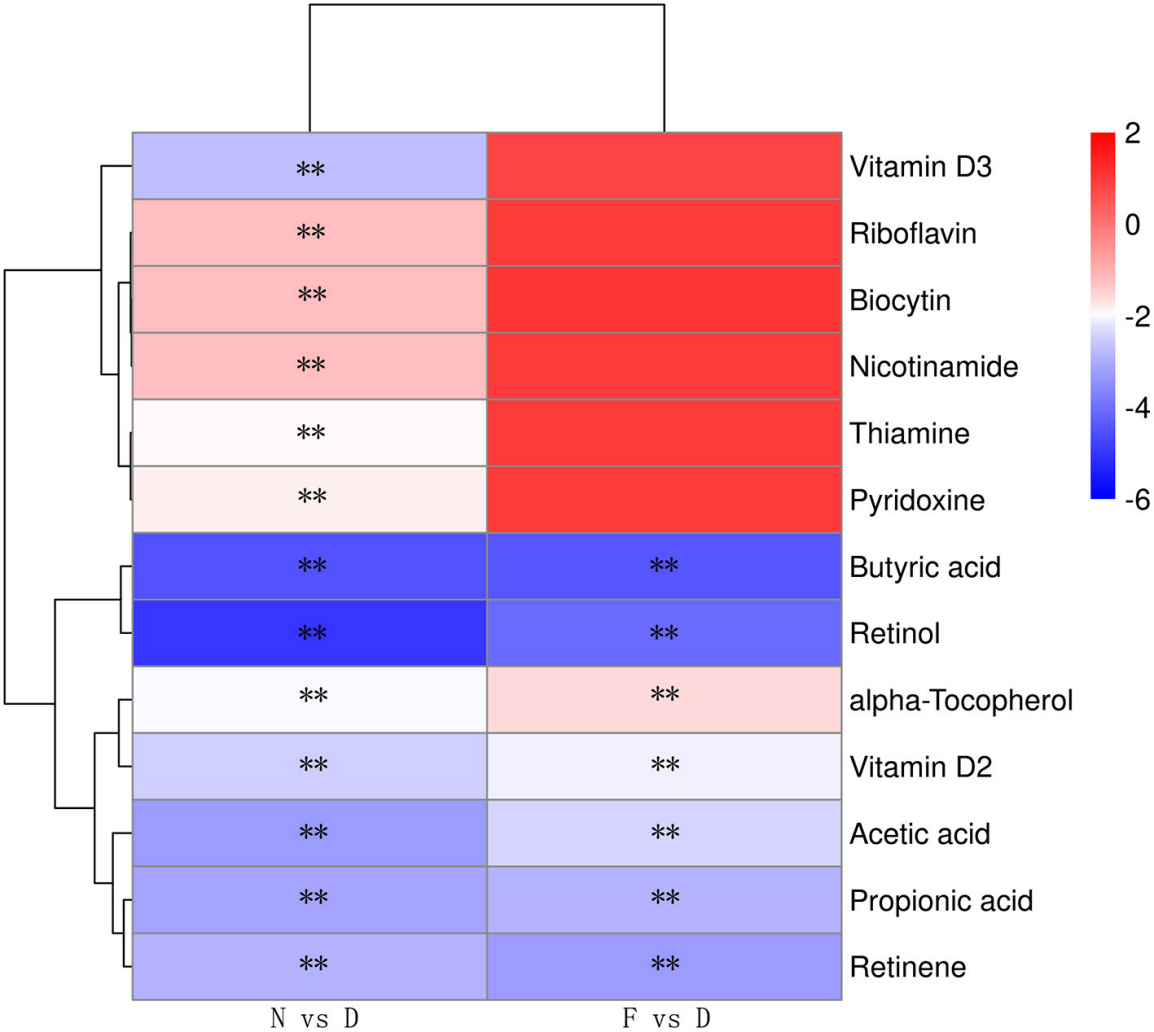
Heatmap of the differential metabolites of vitamins and SCFAs (*n* = 10). Serum samples were collected in the 48 day of age without MG challenge. ^**^Indicates *P* < 0.01.

**Figure 6:**
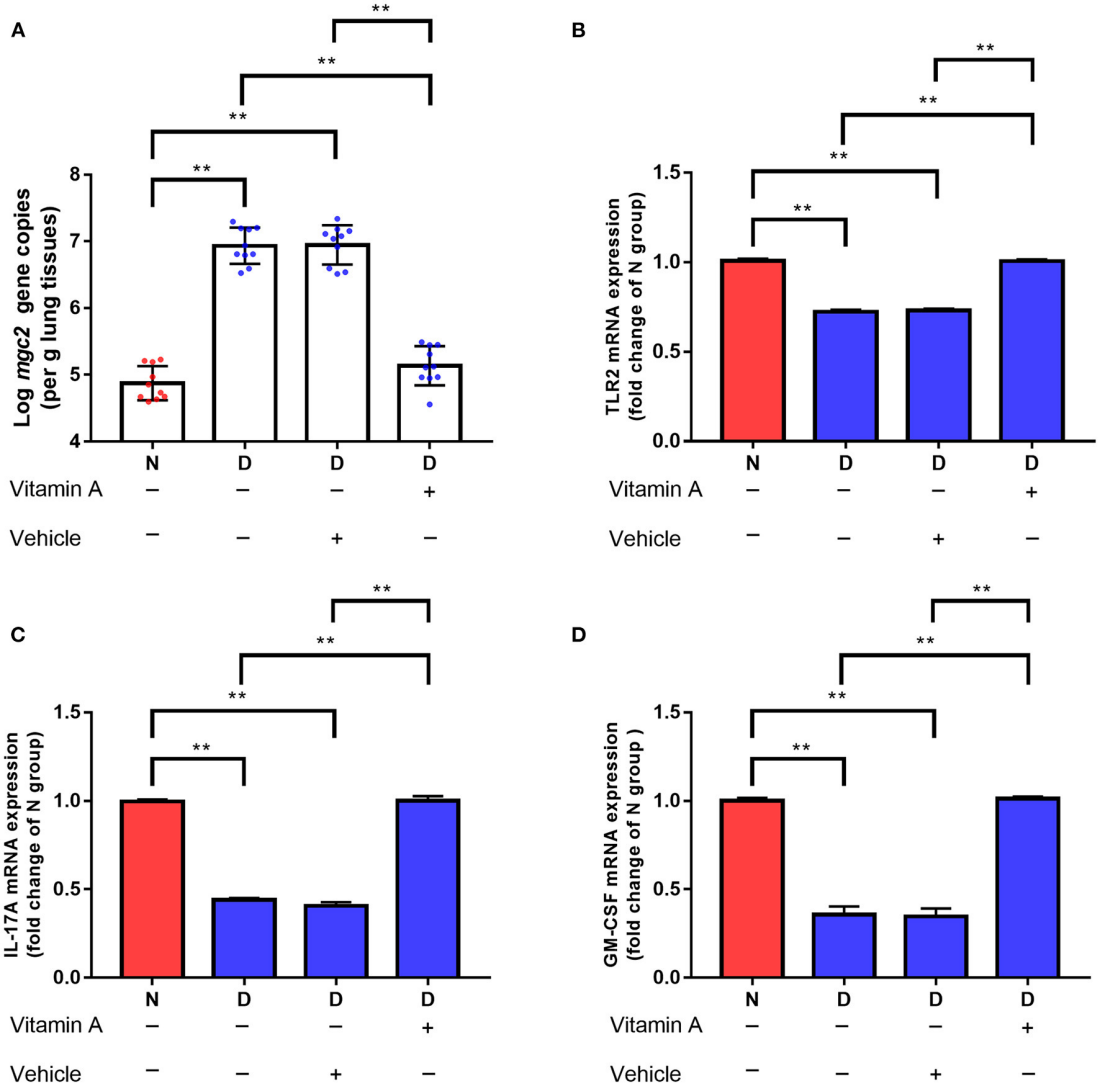
Effects of vitamin A supplementation on MG colonization in lung of gut microbiota dysbiosis group. **(A)** *Mgc2* gene copies in chicken lung after MG infection (*n* = 10). **(B)** TLR2 mRNA expression levels in chicken lung after MG infection (*n* = 6). **(C)** IL-17 mRNA expression levels in chicken lung after MG infection (*n* = 6). **(D)** GM-CSF mRNA expression levels in chicken lung after MG infection (*n* = 6). Indicated groups were orally administered vitamin A at 250 IU/kg feed from day 48 to day 51 showed in Figure 1. ^**^Indicates *P* < 0.01.

The profound influence of gut microbiota on the host is strongly associated with gut microbiota metabolites. Targeted metabolomics results showed that the contents of retinol, retinene and alpha-tocopherol were significantly decreased in the D group compared with the N and F groups (Figure 5, *P* < 0.01). Retinol and retinene both are bioactive molecules of vitamin A, and they can be transformed into each other *in vivo* and have same functions. The alpha-tocopherol is a fat-soluble vitamin that enhances vitamin A absorption. In addition, vitamin A deficiency is associated with *Mycoplasma* infection (22), therefore, vitamin A may be a key metabolite that contributes MG clearance. In D group, oral administration of vitamin A significantly reduced MG colonization in lung (Figure 6A, *P* < 0.01). Consistent with results of MG clearance, oral administration of vitamin A increased TLR2, IL-17 and GM-CSF mRNA expression levels in lung of D group (Figures 6B–D, all *P* < 0.01).

**Figure 7:**
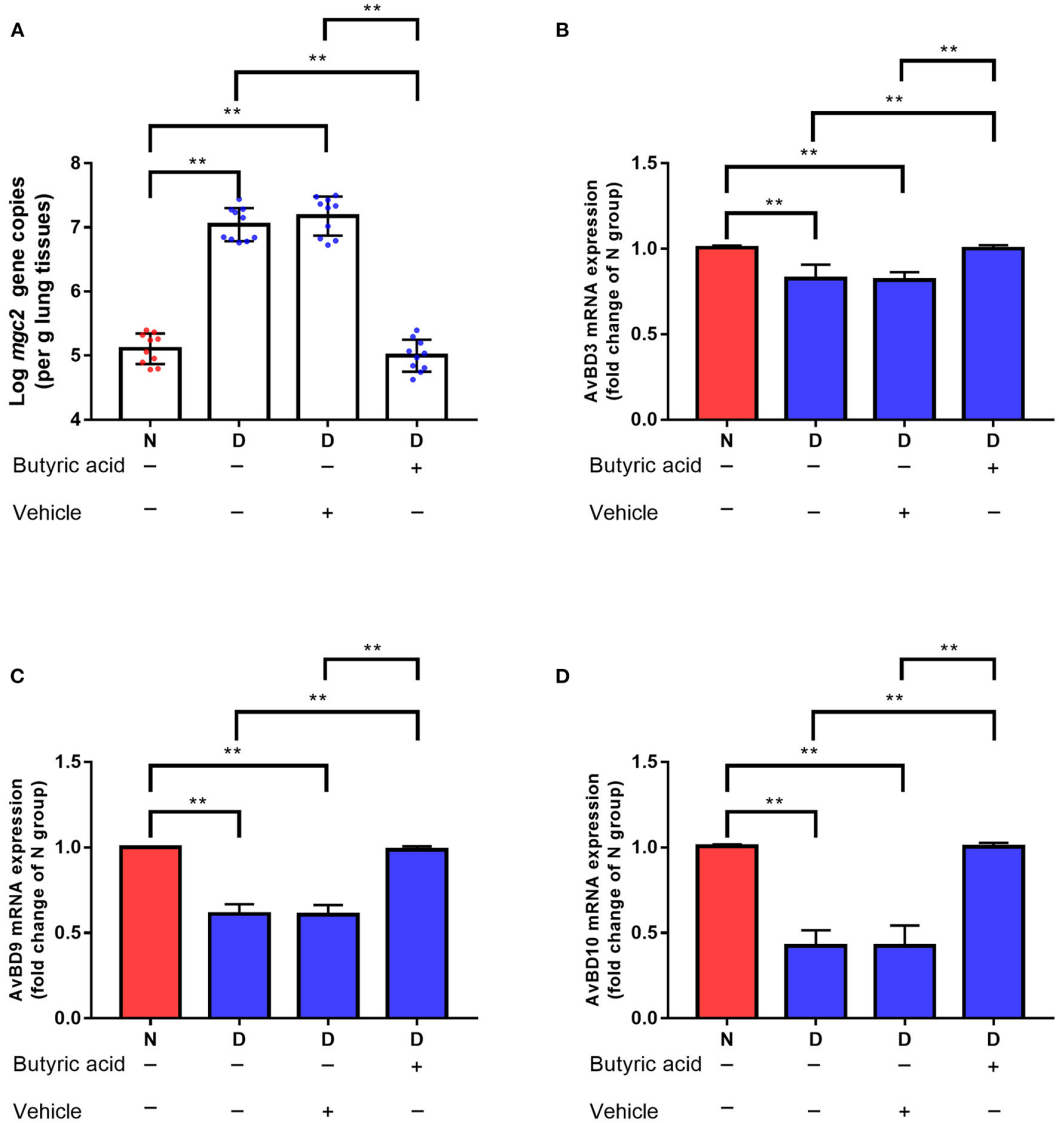
Effects of butyric acid supplementation on MG colonization in lung of gut microbiota dysbiosis group. **(A)** *Mgc2* gene copies in chicken lung after MG infection (*n* = 10). **(B)** AvBD3 mRNA expression levels in chicken lung after MG infection (*n* = 6). **(C)** AvBD9 mRNA expression levels in chicken lung after MG infection (*n* = 6). **(D)** AvBD10 mRNA expression levels in chicken lung after MG infection (*n* = 6). Indicated groups were orally administered sodium butyrate at 1 g/kg feed from day 48 to day 51 showed in Figure 1. ^**^Indicates *P* < 0.01.

Targeted metabolomics results also showed that the contents of SCFAs (acetic acid, propionic acid, and butyric acid) were significantly decreased in the D group compared to the N and F groups (Figure 5, all *P* < 0.01). Since butyric acid decreased the most and was closely related to immune function (23), butyric acid was selected for further verification. In D group, oral administration of butyric acid significantly reduced MG colonization in lung (Figure 7A, *P* < 0.01). Consistent with results of MG clearance, oral administration of butyric acid increased hose defense peptide genes (AvBD3, AvBD9, AvBD10) mRNA expression levels in lung of D group (Figures 7B–D, all *P* < 0.01). Taken together, the above results showed that gut microbiota dysbiosis aggravated MG colonization in lung that may be due to disturbed SCFAs and vitamin A metabolism.

## Discussion

Gut microbiota composition of chickens tends to stabilize at 28 days of age (24), therefore, chickens of this age are selected to carry out gut microbiota-related experiments. In order to elucidate the relationship between gut microbiota and MG infection, we fed four antibiotics to disturb gut microbiota, which was widely used in the study of gut microbiota (4). Previous studies have showed that gut microbiota help the body to resist lung infections caused by *Streptococcus pneumoniae* and *Klebsiella pneumonia* (4, 10). Mice with the normal gut microbiota had about 80% survival rate after infection with influenza virus, while only one-third of mice survived after gut microbiota elimination (25). In the present study, MG colonization in the gut microbiota dysbiosis group was significantly increased compared to normal gut microbiota group, which may be related to the low immune response after microbiota dysbiosis. GM-CSF mRNA expression levels in the gut microbiota dysbiosis group was significantly reduced, the important role of GM-CSF during MG infection was further demonstrated by using rGM-CSF. IL-17 is a crucial cytokine, it has been reported that IL-17 plays a positive role in promoting pathogen clearance by regulating GM-CSF during the innate response to pulmonary *Streptococcus pneumoniae* and *Klebsiella pneumoniae* infection (4). Similarly, this experiment also found that GM-CSF was regulated by IL-17 during MG infection. A variety of cells in the intestine, including T-helper type 17 cells, can produce IL-17, and IL-17 can also be produced in respiratory macrophages and NK cells (4). Therefore, in this experiment, the source of IL-17 and the mechanism of gut microbiota controlling IL-17 production still need to be further studied.

The innate immune response is the first line of defense against pathogens. This rapid, non-specific response depends on the recognition of pathogen recognition receptors. Toll-like receptors (TLRs) are the most widely studied pattern-recognition receptors. The activation of TLRs leads to the activation of downstream signal kinases and transcription factors, which leads to the transcription of encoded inflammatory factor genes and induces innate immune responses (26). Previous study found that TLRs ligands supplementation restored host defenses to *Escherichia coli* and *Klebsiella pneumoniae* infection in the lung (26, 27). In this experiment, intranasally administered TLR2 ligand contributed to MG removal in the lung, indicating that TLR2 played an important role in the initial immune of MG infection. Interestingly, oral administration of TLR4 ligand in this experiment has no effect on MG removal (data not shown), although studies have shown that TLR2 or TLR4 activation are both involved in MG induced inflammatory response (1, 9). However, the role of TLRs in the MG infection process and their relationship with each other need to be further studied. This study only confirms that in the early stage of MG infection, the anti-MG infection effect of gut microbiota depend on TLR2 activation.

Gut microbiota metabolites are considered important mediators of gut-lung interactions. For example, gentamicin induced disorder of gut microbiota results in increased branched-chain amino acids levels that suppress immune cells development which could contribute to enhanced severity of the influenza infection (28). In addition, gut microbiota dysbiosis aggravated lung histopathologic injury, up-regulated pro-inflammatory cytokine production and air-blood permeability, and increased bacterial load caused by avian pathogenic *Escherichia coli*, which partially due to reduced gut microbiota-derived acetate levels (8). In the present study, the levels of SCFAs and vitamin were determined, because SCFAs are important metabolites of gut microbiota, especially butyrate could induce differentiation of macrophages and host defense peptides expression with potent antimicrobial function (23), and vitamin deficiency is associated with severe *Mycoplasma* infection (22). The results confirmed that dysbiosis of the gut microbiota resulted in increased susceptibility of chickens to MG challenge partially result from abnormal intestinal metabolic butyric acid and vitamin levels. In conclusion, we confirmed that gut microbiota provide protection against MG colonization. Novel therapeutic targets that focus on the gut microbiota may be effective in controlling MG infection in poultry.

## Data Availability Statement

The datasets presented in this study can be found in online repositories. The names of the repository/repositories and accession number(s) can be found below: NCBI SRA; PRJNA601545.

## Ethics Statement

The animal study was reviewed and approved by all animal experiments were performed according to the guidelines of Laboratory Animal Ethics Committee of Shanxi Agricultural University (Shanxi, China) in compliance with Laboratory animal-Guideline for ethical review of animal welfare (GB/T35892-2018, National Standards of the People's Republic of China).

## Author Contributions

JW, MI, and JL designed the study and wrote the paper. JW, MI, JL, and XC finished experiments. MI made critical revisions to the paper. All authors contributed to the article and approved the submitted version.

## Funding

This work was supported by the National Natural Science Foundation of China (31973005 and 31772801), the project of Science and Technology Innovation Fund of Shanxi Agricultural University (2021BQ75) and Scientific and Technological Innovation Programs of Higher Education Institutions in Shanxi (STIP, 2021L161).

## Conflict of Interest

The authors declare that the research was conducted in the absence of any commercial or financial relationships that could be construed as a potential conflict of interest.

## Publisher's Note

All claims expressed in this article are solely those of the authors and do not necessarily represent those of their affiliated organizations, or those of the publisher, the editors and the reviewers. Any product that may be evaluated in this article, or claim that may be made by its manufacturer, is not guaranteed or endorsed by the publisher.
